# Improved Calibration Functions of Three Capacitance Probes for the Measurement of Soil Moisture in Tropical Soils

**DOI:** 10.3390/s110504858

**Published:** 2011-05-03

**Authors:** Ali Fares, Farhat Abbas, Domingos Maria, Alan Mair

**Affiliations:** 1 Natural Resources and Environmental Management Department, University of Hawaii-Manoa, Honolulu, HI 96822, USA; E-Mail: farhat@hawaii.edu; 2 School of Engineering and Computer Sciences, New York Institute of Technology, New York, NY 10023, USA; E-Mail: dmaria@nyit.edu; 3 Geology and Geophysics Department, University of Hawaii-Manoa, Honolulu, HI 96822, USA; E-Mail: mair@hawaii.edu

**Keywords:** sensor calibration, single capacitance sensors, soil water content, tropical soils, variable soil properties

## Abstract

Single capacitance sensors are sensitive to soil property variability. The objectives of this study were to: (i) establish site-specific laboratory calibration equations of three single capacitance sensors (EC-20, EC-10, and ML2x) for tropical soils, and (ii) evaluate the accuracy and precision of these sensors. Intact soil cores and bulk samples, collected from the top 20 and 80 cm soil depths at five locations across the Upper Mākaha Valley watershed, were analyzed to determine their soil bulk density (*ρ*_b_), total porosity (*θ*_t_), particle size distribution, and electrical conductivity (EC). Laboratory calibration equations were established using soil packed columns at six water content levels (0–0.5 cm^3^ cm^−3^). Soil bulk density and *θ*_t_ significantly varied with sampling depths; whereas, soil clay content (CC) and EC varied with sampling locations. Variations of *ρ*_b_ and *θ*_t_ at the two depths significantly affected the EC-20 and ML2x laboratory calibration functions; however, there was no effect of these properties on calibration equation functions of EC-10. There was no significant effect of sampling locations on the laboratory calibration functions suggesting watershed-specific equations for EC-20 and ML2x for the two depths; a single watershed-specific equation was needed for EC-10 for both sampling depths. The laboratory calibration equations for all sensors were more accurate than the corresponding default equations. ML2x exhibited better precision than EC-10, followed by EC-20. We conclude that the laboratory calibration equations can mitigate the effects of varying soil properties and improve the sensors’ accuracy for water content measurements.

## 1. Introduction

The water content of surface soils is more dynamic than that of deeper soil layers because of the continuous water loss due to evapotranspiration and the periodical water inputs from rainfall and irrigation events. Variations in water content within the vadose zone are also due to variations in soil texture, *ρ*_b_, *θ*_t_, CC, and EC [1–4].

Soil water content is directly measured with the thermo-gravimetric method and/or indirectly with commercially available soil water content monitoring sensors, *i.e.*, capacitance, TDR and neutron scattering. The thermo-gravimetric method is labor intensive, time consuming, destructive, and discrete for repetitive measurements. Conversely, most of the indirect measurement techniques are logged manually, or in real-time on site with data loggers or remotely via cellular and satellite phones. Details on design, operations, and application of different water content monitoring sensors can be found in Fares and Polyakov [5] and Robinson *et al.* [6]. Site-specific calibration of these sensors is recommended for accurate monitoring of soil water content.

ECH_2_O [7] sensors including EC-5, EC-10 and EC-20, and *ThetaProbe***^®^** [8] sensors including ML2x are single capacitance water content devices, which have been calibrated for different soils in the laboratory [9–11] and in the field [12]. Czarnomski *et al.* [13] tested the EC-20 and found that the default calibration equation under-estimated the actual water content by up to 0.12 cm^3^ cm^−3^, and measurements weren’t sensitive to *ρ*_b_. Overduin *et al.* [14] tested seven different water content sensors, including the ECH_2_O and ML2x ones, for monitoring of the water content of a feather moss stored in different layers. They concluded that the readings of most of the sensors were affected by the spatial variability of the moss bulk density. Logsdon and Hornbuckle [15] compared the performance of ML2x, the updated CS616, and the Stevens Hydra probe. They reported that the larger measurement volume of the CS616 resulted in less spatial variability of its measured water content than that of the ML2x, which has a relatively smaller measurement volume. Foley and Harris [12] assessed the performance of the EC-20 and ML2x in a Black Vertosol from southeast Queensland (Australia) and found considerable over- and under-estimations of water content when using the default calibration equations of these sensors. They also reported a significant impact of *ρ*_b_ on the sensors’ performance and concluded that the site-specific laboratory calibrations can significantly improve the accuracy of both sensors. Bogena *et al.* [16] evaluated the EC-20 and EC-5 (the latest in the ECH_2_O series) in the laboratory and field. They concluded that the sensors’ performance was affected by variability in various soil properties. Mendes *et al.* [17] tested the performance of EC-5 sensors in a pile of poultry manures compacted at five densities (0.32, 0.35, 0.38, 0.42, and 0.47 g cm^−3^). They reported a significant effect of the manure bulk density, temperature, and salinity on sensor performance. Fares *et al.* [11] evaluated the effect of media temperature and salinity on the apparent water content measured with the EC-20. They concluded that ignoring the media temperature and salinity might cause significant errors of up to 0.23 cm^3^ cm^−3^, particularly in the lower water content range.

An ideal sensor, e.g., soil water monitoring sensor, is accurate and precise. Accuracy and precision of most of the water content sensors vary, as does their calibration, with various soil properties [13,18]. Accuracy and precision are the major quantitative assessments of sensor performance [19]; they define how well a sensor’s output represents the actual water content [20]. Accuracy and precision are sometimes incorrectly thought to have the same meaning [21]. Accuracy is the degree of conformity with a standard [22] and; therefore, is the ability of a water content sensor to estimate the actual water content [13]. The root mean square error (RMSE) is a reasonable indicator of a sensor accuracy [23,24]. A smaller RMSE indicates better accuracy.

Precision is an indication of the uniformity or reproducibility of a result and as such relates to the quality of an operation to obtain a result [25]. It is the degree of refinement in the performance of an operation, or the degree of perfection in the instruments and methods used to obtain a result [22]; thus, precision describes the repeatability of a measurement. Precision can also be a measure of the variability of an observation around a statistical true value [23]. Thus, a measure of the precision of an estimate is given by the standard deviation from a true mean [24] or by the variance in the multiple sensor readings simultaneously taken at the same water content level of a uniform medium [21]. Lesser precision is reflected by a larger variance.

**Figure 1.**
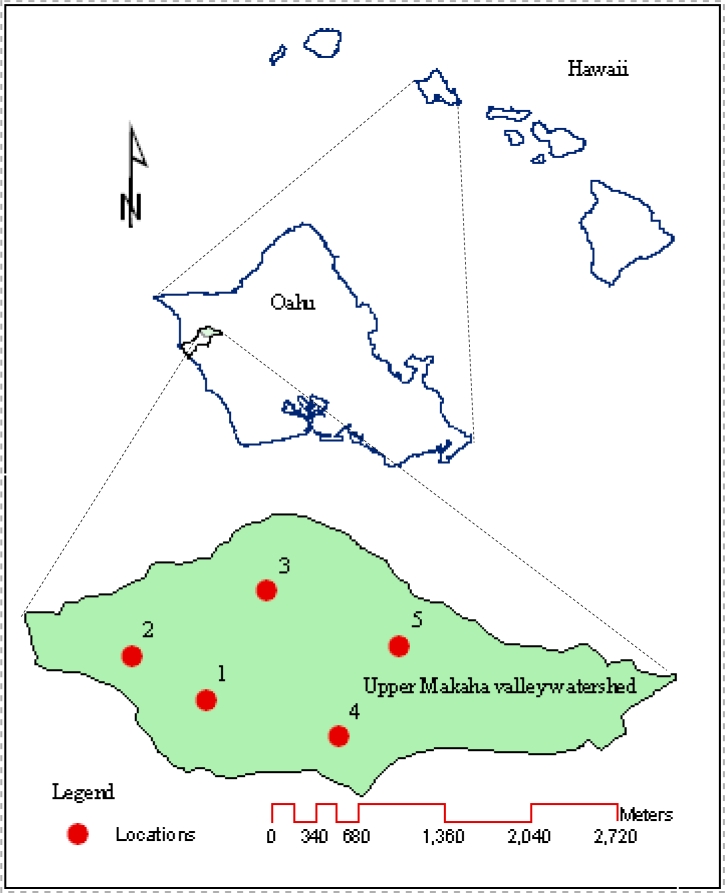
The map of the Upper Mākaha Valley sub-watershed showing the five laboratory calibration study locations where the sensors are installed and from which soil samples were collected and used for this work.

The Upper Mākaha Valley watershed is located on West O’ahu (HI, USA), which is the dry leeward side of this tropical island (Figure 1). The watershed has been home to a long-term hydrologic study aiming at determining the effects of rainfall variability, groundwater pumping, and invasive species on the hydrology of the watershed [26]. The watershed has been instrumented with EC-10, EC-20, and ML2x sensors, and other equipments for real-time monitoring of water budget components including recharge below the root zone, changes in soil water storage within the root zone, and actual evapotranspiration. Our hypothesis was that the varying *ρ*_b_, *θ*_t_, CC, and EC of the watershed soils will affect the performance of these sensors. Therefore, the objectives of this study were to: (i) establish site-specific laboratory calibration equations of EC-10, EC-20, and ML2x for tropical soils and (ii) evaluate the accuracy and precision of these sensors.

## 2. Materials and Methods

### 2.1. The Study Site

The soil samples were taken from five long-term monitoring locations across the Upper Mākaha Valley watershed (Figure 1); we refer to them as locations 1 through 5 from here onward. These locations were chosen to represent the spatial variation of elevation, land cover, soil type and slope across the study area. Two of these locations have weather stations; however, all of them are instrumented with soil water content sensors. Soil water content is also monitored at 20 and 80 cm depths. These locations exhibit spatial variations in topography, soil series, and vegetation cover (Table 1). The soils of the lower valley are less permeable than those along the valley ridges; whereas, those of the upper valley are clay loam, silty loam, and silty clay [27].

### 2.2. Soil Water Content Monitoring Sensors

ECH_2_O (EC-10 and EC-20) sensors operate at 5 MHz frequency [7]; whereas *ThetaProbe***^®^** (the ML2x sensor) operates at 100 MHz frequency [8]. The ECH_2_O sensors measure dielectric constant (*ɛ*) of the surrounding soil media and convert it to a single voltage that ranges between 250 and 1,000 mV, which is related to water content through a linear calibration equation [7]. ML2x generates an electromagnetic signal (at 100 MHz) that extends into the soil by an array of four rods, the impedance of which varies with that of the soil. Soil impedance has two major components: the apparent *ɛ* and the ionic (electrical) conductivity; the operating 100 MHz frequency minimizes the latter; hence, changes in impedance are mainly due to soil’s apparent *ɛ* [8]. There is a linear correlation between water content and the square root of the dielectric constant (√*ɛ*) as determined by the ML2x [28,29]. The configuration of the ML2x makes it less sensitive to minor air gaps and soil variations [15].

### 2.3. Soil Sampling and Analyses

Three replicates of undisturbed soil core samples (radius = 2.5 cm; height = 7.5 cm) were collected with a sludge hammer soil sampler (Soilmoisture Equipment Crop. Santa Barbara, CA, USA) at 20 and 80 cm depths from the five locations (Figure 1). The soil cores were carefully trimmed, sealed with caps, placed in labeled Ziplock plastic bags, and transported in a cooler to the laboratory where the caps from the bottom of the cores were replaced with fine nylon mesh to secure the soil inside the cores. The caps from the top of the cores were removed and the cores were then placed vertically in a tray filled with water for 24 h, letting them slowly saturating from their bottom. The saturated samples were weighed and then oven dried at 105 °C for 48 h and weighed again. The values of *ρ*_b_ and *θ*_t_ were calculated following the procedures described by Grossman and Reinsch [30] and Flint and Flint [31], respectively.

Three replicates of bulk soil samples were also collected from 20 and 80 cm depths at each location. The samples were thoroughly mixed to produce a representative sample for each depth at every location. These samples were air dried and sieved (<2 mm); a sub-sample was used to determine their particle size distribution using the hydrometer method [32]. The textural triangle of the United States Department of Agriculture (USDA) classification scheme was used to determine the soil textural class. These samples were also used to prepare 1:2 soil:water solutions for measurements of EC with the corresponding electrodes connected to a multi-functional *sympHony*^®^ meter (Model SB90M5; Batavia, IL, USA).

### 2.4. Column Preparation for Laboratory Calibrations

Polyvinyl chloride (PVC) cylindrical columns (internal radius = 5 cm; height = 40 cm) were used for the laboratory calibration of the selected sensors. Sieved (<2 mm) and oven-dried (105 °C; 48 h) representative soil bulk samples from the two depths of the five locations were separately packed in these columns. For each location and depth, starting with oven-dried soil, an incremental amount of deionized water was added to and thoroughly mixed with the dry soil to produce soil media of six water content levels (*i.e.*, 0, 0.1, 0.2, 0.3, 0.4, and 0.5 cm^3^ cm^−3^). As incremental amounts of soil were poured in columns during packing, the soil columns were gently tapped from their sides and uniformly compacted from the top to attain the field bulk density of the corresponding depths and locations (Table 2). The ECH_2_O sensors were vertically placed in the center of the columns during packing; whereas, the ML2x sensors were smoothly inserted in the packed columns, which were covered with a tight-fit Styrofoam lid to prevent water evaporation.

The columns filled with soils at the desired water content level with the sensors inserted in them were left for 2 h to attain equilibrium. Ten consecutive readings at 1-minute intervals were logged with data loggers and later used to calculate an average sensor reading for each sensor and for the particular water content. At the end of each calibration experiment, actual water content was determined from soil samples collected near sensor positions in the columns following the thermo-gravimetric method. These laboratory experiments were conducted at a constant room temperature of 22 ± 2 °C.

### 2.5. Data Analyses

Values of actual water content were plotted *versus* the respective readings of the ECH_2_O (mV) and ML2x (√*ɛ*) sensors and linear calibration equations were established separately for the two sampling depths (20 and 80 cm) at every location. A factorial analysis of variance (ANOVA) was conducted to evaluate the effect of sampling depth and location on (i) *ρ*_b_, *θ*_t_, CC, and EC and (ii) the slope and *y*-intercept functions (*a* and *b*) of the laboratory calibration equations using the Statistix software package [33]. The performance of the laboratory calibration equations were evaluated based on P and *r* values obtained from the regression between the calculated and the actual water contents.

Mean bias error (MBE) was used to determine under- and/or over-estimation of water content by the laboratory and default calibration equations. Positive values of MBE indicate over-estimation, whereas negative values indicate under-estimation of water content from their actual values. RMSE was used as an indicator of sensor’s accuracy. Sensor accuracy was assumed very poor, poor, fair, and good for RMSE ≥ 0.1, 0.1 > RMSE ≥0.05, 0.05 > RMSE ≥ 0.01, and RMSE < 0.01 cm^3^ cm^−3^, respectively. MBE (cm^3^ cm^−3^) and RMSE (cm^3^ cm^−3^) were calculated as follows:

(1) $$\text{MBE}=\sum_{i=1}^{n}(\theta_{ci}-\theta_{ai})/n$$

(2) $$\text{RMSE}=\sqrt{\sum_{i=1}^{n}{\left(\theta_{ci}-\theta_{ai}\right)}^{2}/n}$$

where *θ_ci_* and *θ_ai_* are the individual values of calculated and their corresponding actual water contents in cm^3^ cm^−3^, respectively, and *n* is the number of observations. Improvement in the sensor accuracy with the use of laboratory calibration equations over the corresponding default equations was gauged with the percent reduction in RMSE calculated as:

(3) $$\text{Percent}\;\text{reduction}\;\text{in}\;\text{RMSE}=\left(\frac{{\text{RMSE}}_{\text{Def}}-{\text{RMSE}}_{\text{Lab}}}{{\text{RMSE}}_{\text{Def}}}\right)\times 100$$

where RMSE**_Def_** and RMSE**_Lab_** are the RMSE of default and laboratory calibration equations, respectively. Sensor precision was gauged by the variance in the multiple sensor readings that were simultaneously taken from a uniform medium at the same water content level. Larger variance indicates poorer precision.

## 3. Results and Discussion

### 3.1. Soil Properties at Sampling Depths and Locations

**Figure 2.**
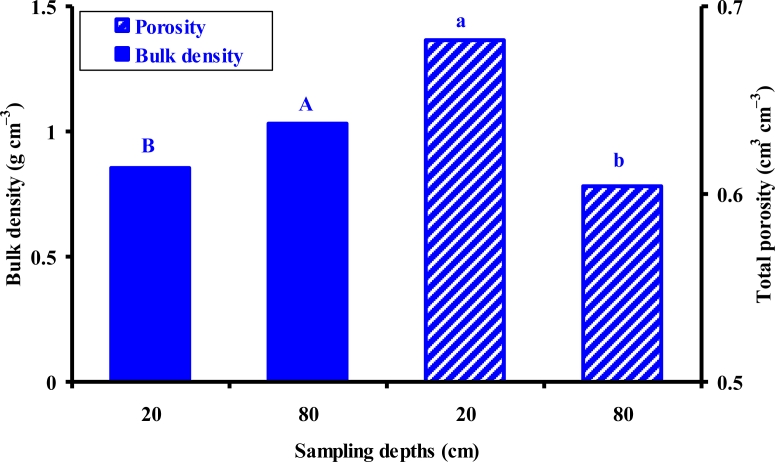
Effect of sampling depths on bulk density and total porosity of soil samples collected from 20 and 80 cm soil depths. Tukey’s mean separation results are shown by the different letters, *i.e.*, the two groups with two different letters were statistically different.

**Figure 3.**
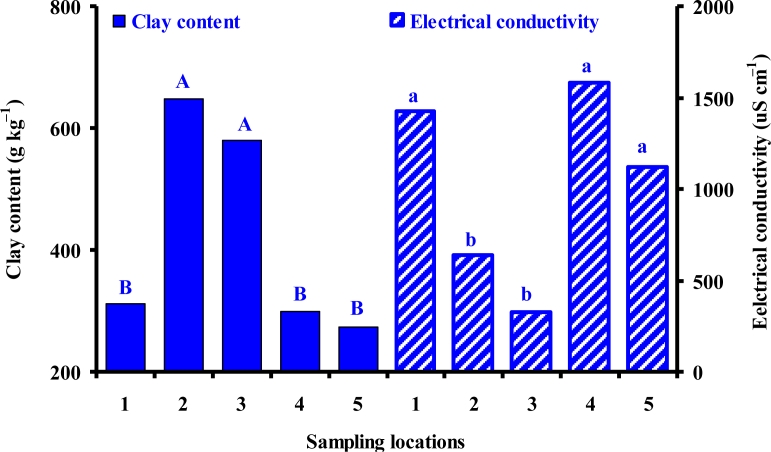
Effect of sampling locations on clay content and electrical conductivity of soil samples collected from locations 1 through 5 across the watershed. Tukey’s mean separation results are shown by the different letters, *i.e.*, the two groups with two different letters were statistically different.

There was a significant increase in *ρ*_b_ (P < 0.05) and a consequent significant decrease in *θ*_t_ (P < 0.05) with increase in soil depth (Table 3). Larger *ρ*_b_ and smaller *θ*_t_ at 80 cm soil depth may be due to compaction from the overburden of the top soil layer. There were statistically significant larger *ρ*_b_ and smaller *θ*_t_ at 80 cm than at 20 cm depth, respectively (Figure 2). At 80 cm depths, *ρ*_b_ ranged between 0.93 and 1.27 g cm^−3^; however, at 20 cm depths, it ranges between 0.69 and 0.95 g cm^−3^ (Table 2). The smaller values of *ρ*_b_ resulted in larger values of *θ*_t_ given their inverse relationship *θ*_t_ = 1 – *ρ*_b_/*ρ*_s_, where *ρ*_s_ is the soil particle density. At 20 cm depth, *θ*_t_ ranged between 0.66 and 0.72 cm^3^ cm^−3^; whereas, at 80 cm depth, it ranged between 0.56 and 0.67 cm^3^ cm^−3^. Sampling location had a highly significant (P < 0.01) effect on CC and a significant (P < 0.05) effect on EC values (Table 3). At locations 2 and 3, the values of CC were significantly larger and those of EC were smaller than those at other locations, respectively (Figure 3). Based on the USDA soil classification method, the soil type at locations 1 and 4 is clay loam (Table 2). Locations 2 and 3 have a clay soil; whereas, location 5 has a loam-sandy loam duplex at 20 and 80 cm depths, respectively.

The values of EC at 20 cm soil depth were almost double of those at 80 cm depth at locations 1 through 3; whereas, at locations 4 and 5, the EC values at 20 cm depth were 1.5 and 1.2 times those at 80 cm depths, respectively (Table 2). Release of nutrients due to decomposition of organic matter from tree litter in these forested watershed soils could be a reason for these larger EC values of the surface (20 cm depth) soil samples. Larger EC values at locations 4 and 5 might be due to the mineral composition of these oxisol and ulitsol that include iron and aluminum oxides, hydroxides, quartz, kaolin, clay minerals, and organic matter. Most tropical soils, including these in the study site, are acidic due to high leaching under warm temperature and intense rainfall conditions [34,35]. There was no significant effect of sampling depth on CC, and EC, and of sampling location on *ρ*_b_ and *θ*_t_ (Table 3).

### 3.2. Laboratory Calibration Equations

The laboratory calibration equations of EC-10, EC-20, and ML2x (Table 4) accurately (P < 0.001; *r* > 0.95) predicted the actual water content (RMSE 0.93 × 10^−2^ to 5.59 × 10^−2^ cm^−3^ cm^−3^) compared with their respective default equations (RMSE 3.69 × 10^−2^ to 10.7 × 10^−2^ cm^−3^ cm^−3^). The values of MBE of the laboratory calibration equations were 10 to 100 times smaller than those of their corresponding default equations. This indicates that the laboratory calibration improved the sensors’ performance. The default calibration equations substantially under-estimated the actual water content compared with the site-specific laboratory calibration equations of the tested sensors.

Under-estimation of the actual water content, either by laboratory or by default calibration equations for ML2x, may be attributed to the high CC [37]. Soils that have high CC contain larger amounts of bound water due to the large surface areas of clay particles compared to silt and sand particles. Bound water, has lower *ɛ* than free water [38] and its proportion in a soil positively correlates with the soil surface area. With the increase of CC in a soil, the proportion of bound water to free water increases. Consequently, the smaller *ɛ* of the bound water in clay soils causes under-estimation of actual water content in high CC soils [39]. This is especially true at low water content where the ratio of bound water to free water substantially increases [18].

### 3.3. Effect of Soil Depth and Location on the Calibration Equations

The slope and *y*-intercept functions (*a* and *b*) of the laboratory calibration equations of the EC-10, EC-20, and ML2x sensors for the two depths and five locations were evaluated for the effect of soil sampling depth and location. There was no statistically significant effect of sampling locations on them; but there was a significant (P < 0.05) effect of soil depths on *a* and *b* of the EC-20 and ML2x equations. The effect of sampling depth on the calibration equations of the EC-20 and ML2x was maybe due to the significant differences (P < 0.05) in the values of *ρ*_b_, *θ*_t_, and CC at the two depths (Tables 2, 3). Huang *et al.* [36] and Foley and Harris [12] also reported that the EC-20 and ML2x were sensitive to varying *ρ*_b_. For the EC-20 and ML2x, there was no significant effect of sampling locations on slope and *y*-intercept functions of the laboratory calibration equations. Therefore, one calibration equation per depth can be used for the entire watershed for each sensor. The sampling depths or the locations did not affect the laboratory calibration equation functions of the EC-10 suggesting that it needs one calibration equation for the entire watershed, irrespective of depth.

### 3.4. Watershed-Specific Calibration Equations

One watershed-specific calibration equation for the EC-10 and two for the EC-20 and ML2x (one for each depth) were established (Table 5). Calculation of the water content using these watershed-specific calibration equations resulted in smaller RMSE and MBE than with their corresponding default equations. The watershed-specific laboratory calibration equation of the EC-10 was more accurate than its corresponding default equation as its MBE is five times smaller than that of the default equation. Similarly, there was an improvement in the accuracy of the EC-20 with the watershed-specific laboratory calibration equations for the two depths. The ML2x watershed-specific laboratory calibration equations for the two depths were more accurate (MBE 0.001 × 10^−2^ and −1 × 10^−6^ cm^−3^ cm^−3^ for 20 and 80 cm, respectively) than the ML2x default equation (MBE −0.565 × 10^−2^ and −2.96 × 10^−2^ cm^−3^ cm^−3^ for 20 and 80 cm, respectively).

**Figure 4.**
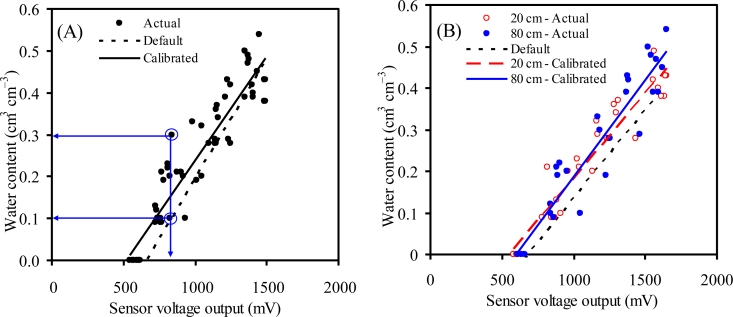

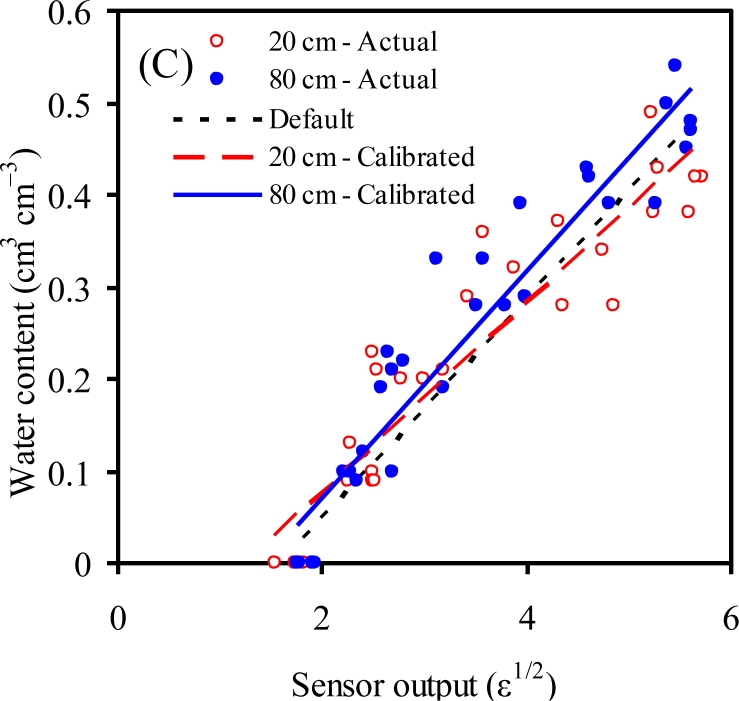
Actual soil water content as a function of the readings of the EC10 (**A**), EC-20 (**B**), and ML2x (**C**) using soil samples from 20 and 80 cm depths across the watershed. Arrows connecting actual water content data points (circled blue) with *x* and *y* axes in 4A show an example of absolute error from EC-10 as it gave same reading (*ca.* 830 mV) for 0.1 and 0.3 cm^3^ cm^−3^ water contents resulting in 20% error.

The accuracy of the laboratory calibration equations for ECH_2_O sensors was the highest for the water content between 0.2 and 0.5 cm^3^ cm^−3^ [Figure 4(A,B)]. There was slight decrease in the accuracy of the watershed-specific laboratory calibration equations of the EC-20 at 20 and 80 cm depths for the water content ≤ 0.2 cm^3^ cm^−3^ [Figure 4(B)]. For the ML2x, the laboratory calibration equations performed better at water content ≥ 0.25 cm^3^ cm^−3^ [Figure 4(C)]. However, there was no difference between the two calibration equations of ML2x for water content ≤ 0.15 cm^3^ cm^−3^ [Figure 4(C)]. Overall, the default calibration equations of the ML2x performed better than those of the ECH_2_O sensors across the tested range of water content. The EC-10 had the highest variation in its readings among the three sensors; it had an absolute error as high as 20% as shown in Figure 4(A), 830 mV corresponds to 0.10 and 0.30 cm^3^ cm^−3^ actual water contents. The EC-20 and ML2x showed absolute errors of up to 10 to 12% and 10 to 15%, respectively, especially at medium and high soil water content ranges [Figure 4(B,C)].

### 3.5. Sensor Accuracy and Precision

Significant variations of *ρ*_b_ and *θ*_t_ across the sampling depths and of CC and EC across the sampling locations had a significant effect on sensors’ accuracy (large RMSE values in Tables 4 and 5). The EC-20 exhibited poor accuracy with laboratory (RMSE 5.59 × 10^−2^ cm^3^ cm^−3^) and default (RMSE 6.27 × 10^−2^ cm^3^ cm^−3^) calibration equations at 80 cm depth of location 1 which may be due to the large value of *ρ*_b_, *i.e.*, 1.27 g cm^−3^ (Table 3). Likewise,a small value of *ρ*_b_, *i.e.*, 0.69 g cm^−3^ at 20 cm depth of location 5 resulted in a fair accuracy of the EC-20 with the laboratory (RMSE 2.45 × 10^−2^ cm^3^ cm^−3^) and default (RMSE 4.76 × 10^−2^ cm^3^ cm^−3^) calibration equations.

The accuracy of the EC-10 sensor was fair with the use of laboratory calibration equation and poor with the use of default equation, except at the two depths of location 1 and at the 20 cm depth of location 5 where the default equation had similar accuracy to that of laboratory equations. Overall, the laboratory calibration equations improved the accuracy of the tested sensors as compared to their corresponding default equations except under large value of *ρ*_b_ at 80 cm of locations 1 (EC-20; *ρ*_b_ 1.27 g cm^−3^) and 2 (EC-10; *ρ*_b_ 1.06 g cm^−3^). The use of default calibration equations of all sensors resulted in poor to very poor sensor accuracy especially at locations 2 and 3 (Table 4); the sensors’ accuracy varied between fair and poor at the remaining locations.

The percent reduction in RMSE, calculated from Equation 3, reflected that the laboratory calibration equations of each sensor improved their accuracy compared with the use of their corresponding default equations (Table 6). The percent reduction in RMSE, calculated from Equation 3, reflected that the accuracy of each sensor improved with the use of their laboratory calibration equations compared with the corresponding default equations (Table 6). Percent reduction in RMSE ranged from 11 to 89% for EC-20, 4.7 to 64% for the EC-10, and 7 to 47% for the ML2x. These results reflect improvement in the sensors’ accuracy with the use of laboratory calibration equations over their corresponding default equations in the ascending order EC-20 > EC-10 > ML2x.

The watershed-specific calibration equations also improved the accuracy of the EC-10 by 25%; whereas, the accuracy of the EC-20 and ML2x was improved by 32 and 44% for 20 cm depth and by 7.5 and 14% for 80 cm soil depth. Jones *et al.* [40] attributed the poor performance of ECH_2_O sensors to EC effects. Such effects dominate the output of sensors that operate at frequencies < 100 MHz [41], *i.e.*, ECH_2_O sensors, which operate at 5 MHz. Poor performance of the EC-20 can also be attributed to the sensor’s functionality of averaging its readings over its plane of interface with the soil (*i.e.*, 20 cm) than the EC-10 (*i.e.*, 10 cm).

**Figure 5.**
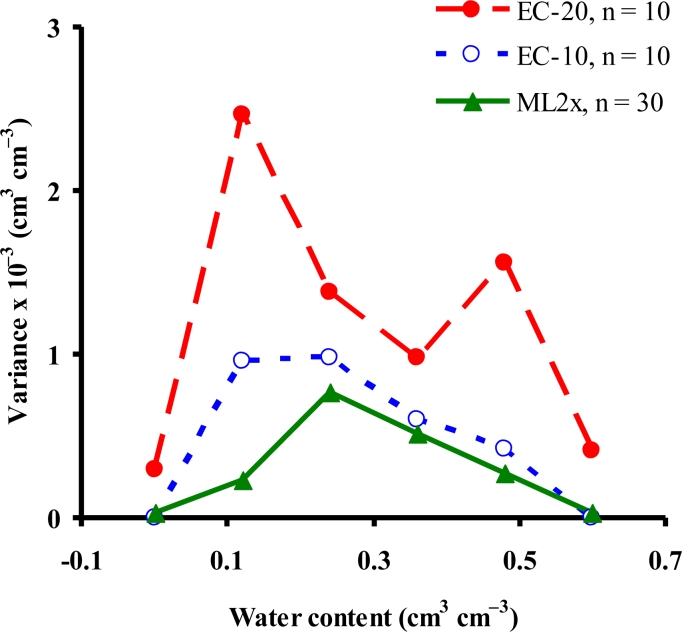
Precision of the EC-10, EC-20, and ML2x sensors judged from the variance of sensor’s readings repeatedly (n = 10 for the ECH_2_O sensors; and n = 30 for the ML2x, where n is number of repeated measurements) taken at the same water content level.

The ML2x exhibited better precision (mean variance; MV 0.31 × 10^−3^ cm^3^ cm^−3^) than the EC-10 (MV 0.49 × 10^−3^ cm^3^ cm^−3^) and EC-20 (MV 1.18 × 10^−3^ cm^3^ cm^−3^) (Figure 5). The variance from the true mean of the actual water content ranged from 0.03 × 10^−3^ to 0.77 × 10^−3^, 0 to 0.98 × 10^−3^, and 0.29 × 10^−3^ to 2.47 × 10^−3^ cm^3^ cm^−3^ for the ML2x, EC-10, and EC-20, respectively. The high precision of the ML2x was may be due to: (i) its rods’ perfect contact with the surrounding media and (ii) its impedance of 100 MHz sinusoidal signal, which is supposed to minimize the effect of EC on sensor’s readings [8,41]. Overall, the sensors showed better precision at low (<0.1 cm^3^ cm^−3^) and high water content (>0.5 cm^3^ cm^−3^) levels than at medium water content (0.15–0.45 cm^3^ cm^−3^) where all the sensors’ exhibited poorer precision. Rosenbaum *et al.* [42] reported higher variances (low precision) than our results from the repeatability experiments of the ECH_2_O and other sensors. Extensively repeated measurements might result in more realistic variances and better representation of sensors’ precision.

## 4. Summary and Conclusions

Laboratory calibration equations of The EC-10, EC-20, and ML2x sensors were established and evaluated for accurate measurement of water content at 20 and 80 cm depths across five locations of the forested Upper Mākaha Valley watershed soils of varying *ρ*_b_, *θ*_t_, CC, and EC. None of the studied soil properties, except CC and EC, was significantly affected by sampling locations. Bulk density and *θ*_t_ significantly varied with sampling depth and consequently affect the laboratory calibration equation functions of the EC-20 and ML2x; however, the calibration equation functions of the EC-10 had no effect of spatial variability neither as a function of depth nor location. Consequently, the EC-10 needed one calibration equation for the entire watershed, irrespective of the soil depths. However, one calibration equation for the entire watershed per depth was needed for EC-20 and ML2x to capture the spatial variations encountered on this tropical watershed. The laboratory calibration equations improved the sensors’ measurement ability as compared to that with their corresponding default equations. The maximum improvement was for the EC-20, followed by the EC-10 and ML2x. Moreover, the ML2x exhibited the highest precision, most probably due to its higher operating frequency, followed by the EC-10 and EC-20. These results reinforce the need for site-specific calibration equations specifically for fields with large spatial variability.

## Figures and Tables

**Table 1.** The elevation, NRCS soil series, and the vegetation of the five monitoring locations.

**Location**	**Elevation, m**	**NRCS soil series**	**Vegetation**
1	343	Mollisol	Christmas berry (*Lycium carolinianum*)
2	477	Inceptisol	Strawberry guava (*Psidium cattleianum*)
3	538	Inceptisol	Ohia (*Metrosideros polymorpha*), Strawberry guava
4	601	Oxisol/Ultisol	Coffee (*Coffea arabica*), Llama (*Artiodactyla camelidae*), Strawberry guava
5	609	Oxisol/Ultisol	Ohia, Strawberry guava, Uluhe (*Dicranopteris linearis*)

**Table 2.** The USDA soil classification, particle size distribution, bulk density (*ρ*_b_), total porosity (*θ*_t_), and the values of electrical conductivity (EC) of the soil samples collected from 20 and 80 cm depths at the five monitoring locations in the Upper Mākaha Valley watershed.

**Location**	**Depth cm**	***ρ*_b_g cm^−3^**	***θ*_t_cm^3^cm^−3^**	**Clay g kg^−1^**	**Sand**	**USDA Soil texture**	**EC^#^μS cm^−1^**
1	20	0.93	0.69	313	269	Clay loam	2,016
	80	1.27	0.56	313	269	Clay loam	840
2	20	0.86	0.67	610	308	Clay	802
	80	1.06	0.60	690	250	Clay	480
3	20	0.83	0.67	521	167	Clay	426
	80	0.97	0.58	640	250	Clay	222
4	20	0.95	0.66	313	218	Clay loam	1,888
	80	0.93	0.67	288	320	Clay loam	1,270
5	20	0.69	0.72	263	421	Loam	1,220
	80	0.93	0.61	288	661	Sandy loam	1,024

# Electrical conductivity measurements were made on 1:2 soil:water solutions.

**Table 3.** Values of the probability (P) and significance levels obtained from the factorial general analysis of variance for bulk density (*ρ*_b_), porosity (*θ*_t_), clay content (CC), and electrical conductivity (EC) as a function of soil depths and sampling locations.

**Factors**	***ρ*_b_**	***θ*_t_**	**CC**	**EC**
Depth	0.0393*	0.0320*	NS	NS
Location	NS	NS	0.0021**	0.0480*
Interaction	NS	NS	NS	NS

*: significant;

**: highly significant; NS: not significant.

**Table 4.** Calibration functions of EC-10, EC-20, and ML2x laboratory calibration equations and statistical indicators for their accuracy in estimating actual water content. The values of RMSE and MBE in parentheses are from the comparison of actual water content with that calculated with the manufacturer calibration equations.

**Station**	**Depth**	**Sensor**	***a***	***b***	**P**	***r***	**RMSE**	**MBE**
	
			**cm^−3^cm^−3^× 10^−2^**				**cm^−3^cm^−3^× 10^−2^**	
1	20	EC-10	0.045	26.020	1.6E-04	0.99	2.18 (4.70)	−0.010 (1.390)
		EC-20	0.040	23.487	7.0E-04	0.98	3.13 (3.95)	0.033 (−2.167)
		ML2x	10.83	14.550	2.4E-03	0.96	4.25 (4.55)	−0.030 (−0.787)
	80	EC-10	0.062	23.090	1.4E-03	0.97	4.42 (4.64)	0.016 (0.008)
		EC-20	0.051	39.035	3.6E-04	0.95	5.59 (6.27)	−0.041 (−5.08)
		ML2x	14.63	28.512	3.5E-05	0.99	1.77 (3.81)	0.000 (−5.44)

2	20	EC-10	0.056	−27.669	1.5E-03	0.99	2.60 (9.67)	−0.026 (−9.312)
		EC-20	0.041	−29.963	6.7E-05	0.99	0.93 (8.49)	0.041 (−7.960)
		ML2x	12.96	−14.989	7.2E-03	0.97	4.37 (8.58)	0.001 (−7.260)
	80	EC-10	0.061	−32.060	3.6E-03	0.95	5.25 (10.7)	0.043 (−9.312)
		EC-20	0.055	−34.963	1.7E-05	0.99	1.40 (8.90)	0.031 (−7.877)
		ML2x	13.44	−16.906	4.0E-03	0.95	5.43 (9.08)	−0.002 (−7.037)

3	20	EC-10	0.051	−24.630	2.6E-03	0.96	4.34 (8.60)	−0.012 (−7.227)
		EC-20	0.043	−21.547	2.6E-03	0.96	4.33 (9.55)	−0.019 (−8.510)
		ML2x	10.39	−11.833	4.2E-03	0.95	4.93 (5.73)	0.000 (−2.067)
	80	EC-10	0.060	−31.592	5.8E-04	0.98	3.43 (9.61)	0.012 (−8.893)
		EC-20	0.053	−31.423	1.3E-04	0.99	2.35 (10.1)	−0.026 (−9.153)
		ML2x	12.43	−16.718	1.2E-03	0.97	4.07 (5.83)	0.001 (−4.113)

4	20	EC-10	0.042	−22.701	2.3E-04	0.99	2.38 (5.82)	0.042 (1.002)
		EC-20	0.037	−21.342	7.9E-04	0.98	3.25 (4.18)	−0.011 (−1.740)
		ML2x	9.072	−13.183	6.7E-03	0.97	3.35 (6.14)	0.000 (4.186)
	80	EC-10	0.047	−24.557	5.4E-04	0.98	2.97 (5.16)	0.018 (2.800)
		EC-20	0.040	−21.146	1.5E-03	0.97	3.83 (6.29)	−0.114 (−4.895)
		ML2x	10.55	−13.378	2.5E-03	0.96	4.36 (4.82)	0.000 (0.860)

5	20	EC-10	0.049	−26.874	3.6E-04	0.98	2.45 (4.44)	−0.018 (−3.012)
		EC-20	0.043	−25.819	3.4E-04	0.99	2.40 (4.76)	−0.011 (−4.090)
		ML2x	10.70	−17.344	1.8E-03	0.99	1.94 (3.69)	−0.348 (2.347)
	80	EC-10	0.057	−31.216	7.6E-04	0.98	3.52 (7.48)	−0.025 (−6.603)
		EC-20	0.048	−28.525	1.3E-03	0.97	4.05 (8.38)	0.024 (−7.048)
		ML2x	12.18	−17.743	7.9E-04	0.98	3.56 (4.23)	0.001 (−2.248)

*a* = slope of calibration equation; *b* = *y*-intercept of calibration equation; P and *r*: probability and coefficient of correlation values obtained from the regression between the calculated and the actual water contents.

**Table 5.** Calibration functions of the EC-10, EC-20, and ML2x site-specific laboratory calibration and default equations and statistical indicators for their accuracy in estimating actual water content.

**Sensor / Calibration**	**Depth, cm**	***n***	***a***	***b***	**RMSE**	**MBE**

			**cm^−3^cm^−3^× 10^−2^**		**cm^−3^cm^−3^× 10^−2^**	
EC-10	20–80	59	0.0496	−25.654	5.54	−0.643
Default	20–80	59	0.0571	−37.597	7.40	−4.390

EC-20	20	29	0.0408	−22.418	4.43	0.009
	80	30	0.0467	−27.942	5.39	0.026
Default	20	29	0.0424	−28.997	6.55	−4.79
	80	30	0.0424	−28.997	8.12	−5.90

ML2x	20	28	10.302	−12.961	5.54	−0.001
	80	30	12.272	−17.357	5.04	−1E–04
Default	20	28	11.900	−19.050	5.99	−0.565
	80	30	11.900	−19.050	5.87	−2.96

*a* = slope of calibration equation; *b* = *y*-intercept of calibration equation.

**Table 6.** Percent improvement in the accuracy of the EC-10, EC-20, and ML2x sensors based on the root mean square error (RMSE) values of the laboratory and default calibration equations to predict the actual soil water content.

**Location**	**Depth, cm**	**EC-10**	**EC-20**	**ML2x**
1	20	54	21	7
	80	4.7	11	54
2	20	73	89	49
	80	51	84	40
3	20	50	55	14
	80	64	77	30
4	20	59	22	45
	80	42	39	10
5	20	45	50	47
	80	53	52	16
Watershed scale	20	25	32	7.5
1–5	80	-	34	14
